# Safety of OnabotulinumtoxinA in the [management of] chronic migraine in pregnancy

**DOI:** 10.3389/fpain.2022.967580

**Published:** 2022-08-18

**Authors:** Liza Smirnoff

**Affiliations:** Headache Division, Stanford Medicine, Palo Alto, CA, United States

**Keywords:** chronic migraine, OnabotulinumtoxinA, pregnancy, headache, women's health

## Background

OnabotulinumtoxinA is an irreversible acetylcholinesterase inhibitor and a neuromuscular blocking agent first approved for use by the FDA in 1989, and most recently updated in 2010 (1). It is currently approved for the management of chronic migraine, defined by the FDA insert as 15 or more headache days per month, each lasting 4 or more hours, and by the ICHD-3 as 15 or more headache days per month, with 8 or more of those days consistent with migraine (1, 2).

To explore the possible implications of using OnabotulinumtoxinA for pregnant patients with chronic migraine, we must first explore the known risks. In terms of known risks, the FDA insert carries a black box warning from postmarketing surveillance, indicating that OnabotulinumtoxinA may spread to surrounding areas to produce symptoms consistent with botulinum toxin effect, including weakness of skeletal and smooth muscles, ultimately describing reports of death and risk highest in children treated for spasticity. However, as noted later in the document, in the specific indication of use for chronic migraine, there are no known definitive serious adverse events reported in either clinical studies or postmarketing surveillance. Specific adverse reactions listed for the indication of chronic migraine included neck pain, headache, worsening migraine, muscular weakness, and eyelid ptosis.

In terms of determining risk, the FDA currently assigns a pregnancy rating of C for OnabotulinumtoxinA, indicating a lack of adequate and well-controlled studies in pregnant women. In original animal studies noted on the FDA insert, intramuscular administration of OnabotulinumtoxinA to pregnant rats during organogenesis produced decreased fetal weight and decreased fetal bone ossification at high doses only at 4 units/kg, which is, approximately, the equivalent of 1½ times the dosing for the average high human dosing for upper limb spasticity (360 units).

In notable human studies, a 24-year review of the Allergan safety database found 574 pregnancies with known OnabotulinumtoxinA exposure (3). No maternal or fetal cases of botulism were reported, and the fetal defect prevalence rate was consistent with that in the general population at 2.7%. Another prospective study looking at the use of OnabotulinumtoxinA for the management of chronic migraine in 45 pregnant patients reported no impact on pregnancy outcomes (4). Additionally, clinical case reports of women affected by botulism illness did not show adverse effects on the pregnancy, including one case where the only notable movement in the patient was the fetus, while the mother was affected by paralysis (5–10). It is therefore reasonable to assume that, if the naturally occurring botulinum toxin, weighing 150 kDA, does not cross the placental barrier, then the complexed OnabotulinumtoxinA molecule which weighs 900 kDA is even less likely to.

Another notable consideration for the use of OnabotulinumtoxinA in the management of chronic migraines during pregnancy is the relative lack of safety of other commonly used preventative medications. Memantine and cyproheptadine alone are listed as Category B for the preventative management of migraines, while the more efficacious beta blockers, SNRI's, and amitriptyline are listed as Category C, and topiramate, valproic acid, and nortriptyline are all listed as Category D (11). In addition to reported risks, the oral absorption and circulation of these compounds are indisputable in comparison to OnabotulinumtoxinA, which may cross into the circulation but is only administered on a quarterly basis.

In all it is a molecule too large to cross the placental barrier (12, 13), and human studies thus have not shown any increase in worsening pregnancy outcomes with its use across a variety of indications in pregnant patients. Likewise, when following the PREEMPT protocol for chronic migraine (14), it is not applied in areas that could compromise respiration or cause significant weakness in the mother. Additionally, although animal studies show some negative outcomes, they are seen only in doses that far surpass those used in chronic migraine. Furthermore, given the lack of safety of most other medications commonly used for migraine during pregnancy, when used appropriately, migraine therapy with OnabotulinumtoxinA may actually reduce the use of other more potentially teratogenic compounds, as well as reduce the migraine-associated disability and potential fetal harm caused by uncontrolled pain. Based on the current data available, OnabotulinumtoxinA remains a very strong option in the management of chronic migraine in pregnancy, with the potential to significantly reduce migraine-related disability and pain and improve the quality of life of our pregnant patients. Additionally, given currently limited observational studies on the use of OnabotulinumtoxinA in pregnant patients, further larger studies looking at long-term outcomes are needed to ascertain both the safety and efficacy of this medication, which has been seen clinically.

## Author contributions

The author confirms being the sole contributor of this work and has approved it for publication.

## Conflict of interest

The author declares that the research was conducted in the absence of any commercial or financial relationships that could be construed as a potential conflict of interest.

## Publisher's note

All claims expressed in this article are solely those of the authors and do not necessarily represent those of their affiliated organizations, or those of the publisher, the editors and the reviewers. Any product that may be evaluated in this article, or claim that may be made by its manufacturer, is not guaranteed or endorsed by the publisher.
